# Is systemic inflammation a missing link between cardiometabolic index with mortality? Evidence from a large population-based study

**DOI:** 10.1186/s12933-024-02251-w

**Published:** 2024-06-20

**Authors:** Bin Xu, Qian Wu, Rui La, Lingchen Lu, Fuad A. Abdu, Guoqing Yin, Wen Zhang, Wenquan Ding, Yicheng Ling, Zhiyuan He, Wenliang Che

**Affiliations:** 1 Department of Cardiology, Shanghai Tenth People’s Hospital, Tongji University School of Medicine, 301 Yanchang Road, Shanghai, 200072 China; 2 Department of Cardiology, Zhongshan-Xuhui Hospital, Shanghai Xuhui Central Hospital, Fudan University, Shanghai, China; 3 Department of Orthopedic Surgery, Orthopedic Institute, The First Affiliated Hospital of Soochow University, 188 Shizijie Road, Suzhou, 215006 Jiangsu China; 4 Research Institute of Clinical Medicine, Jeonbuk National University Medical School, Jeonju, Republic of Korea; 5 Department of Pediatric Surgery and Rehabilitation, Kunshan Maternity and Children’s Health Care Hospital, Kunshan, Jiangsu China

**Keywords:** Cardiometabolic index, Cardiovascular disease, Inflammation, Mortality, National Health and Nutrition Examination Survey

## Abstract

**Background:**

This study sought to elucidate the associations of cardiometabolic index (CMI), as a metabolism-related index, with all-cause and cardiovascular mortality among the older population. Utilizing data from the National Health and Nutrition Examination Survey (NHANES), we further explored the potential mediating effect of inflammation within these associations.

**Methods:**

A cohort of 3029 participants aged over 65 years old, spanning six NHANES cycles from 2005 to 2016, was enrolled and assessed. The primary endpoints of the study included all-cause mortality and cardiovascular mortality utilizing data from National Center for Health Statistics (NCHS). Cox regression model and subgroup analysis were conducted to assess the associations of CMI with all-cause and cardiovascular mortality. The mediating effect of inflammation-related indicators including leukocyte, neutrophil, lymphocyte, systemic immune-inflammation index (SII), neutrophil to lymphocyte ratio (NLR) were evaluated to investigate the potential mechanism of the associations between CMI and mortality through mediation package in R 4.2.2.

**Results:**

The mean CMI among the enrolled participants was 0.74±0.66, with an average age of 73.28±5.50 years. After an average follow-up period of 89.20 months, there were 1,015 instances of all-cause deaths and 348 cardiovascular deaths documented. In the multivariable-adjusted model, CMI was positively related to all-cause mortality (Hazard Ratio (HR)=1.11, 95% CI=1.01-1.21). Mediation analysis indicated that leukocytes and neutrophils mediated 6.6% and 13.9% of the association of CMI with all-cause mortality.

**Conclusion:**

Elevated CMI is positively associated with all-cause mortality in the older adults. The association appeared to be partially mediated through inflammatory pathways, indicating that CMI may serve as a valuable indicator for poor prognosis among the older population.

**Supplementary Information:**

The online version contains supplementary material available at 10.1186/s12933-024-02251-w.

## Background

Cardiometabolic disease, initially conceptualized as a constellation of metabolic dysfunctions heightening the risk for cardiovascular disease (CVD) and diabetes mellitus (DM), stands as a principal cause of death and disability globally [1]. Within the US, expenditures on CVD and DM healthcare have reached $89.3billion and $111.2billion respectively, ranking as the fourth and third highest healthcare costs and exerting considerable pressure on health care resources [2]. Besides, CVD and DM are highly prevalent health conditions in the older population [3]. In the US, the prevalence of CVD and DM are over 78% for those aged>60 years and over 26.8% for those aged>65 years respectively [4, 6]. In 2019, CVD and DM were ranked first and fifth in leading factors that affect life span in age groups over 75 years [7]. Consequently, the identification of modifiable risk factors within the older population, especially in cardiometabolic disease, is imperative for the advancement of global public health and the formulation of preventive strategies.

The pathophysiology of CVD and DM is intricately linked to chronic inflammation, which is implicated in the exacerbation of long-term complications and worse prognosis [8]. Chronic, low-grade inflammation is a significant contributor to insulin resistance and hyperglycemia, precipitating DM and its associated microvascular and macrovascular complications [9, 10]. Meanwhile, inflammation has been considered a key factor in atherosclerosis, thereby accelerating CVD progression [11, 12]. In the older population, chronic, low-grade, systemic inflammation develops with age [11]. Elevated level of inflammation is predictive of all-cause mortality regardless of other established risk factors in the older adults [13, 14]. Therefore, systemic inflammation may have a potential role in mediating the long-term prognosis of the older population.

Cardiometabolic index (CMI), as a metabolism-related index, was an indicator initially devised to forecast DM risk [15]. With the in-depth clinical application, numerous studies demonstrated that CMI was positively associated with risks of CVD and metabolic syndrome (MetS) [16, 18]. Further, research by Jovanovic et al. revealed that adherence to an anti-inflammatory diet correlates with lower CMI and reduced levels of inflammatory indicators [19]. Individuals with high CMI may experience elevated systemic inflammation, potentially aggravating CVD and DM and escalating the risk of long-term complications, leading to a worse prognosis and higher mortality. To our knowledge, there is no prior study to assess the prognostic value of CMI in the older population.

Therefore, our study aimed to evaluate the associations of CMI with all-cause and cardiovascular mortality in the older adults, and further examine whether inflammatory indicators have a potential role in mediating these associations.

## Methods

### Study design and population

The present study is a longitudinal cohort study and the database was from NHANES. NHANES serves as a comprehensive survey designed to amass data on the health status of the United States population. Employing a stratified multistage random sampling methodology, NHANES ensures the representation of a national sample [20]. NHANES was granted approval from the ethical review board of The National Center for Health Statistic, with each participant providing informed consent via signed agreements [21]. The datasets, replete with thorough documentation and protocols, are publicly available on the NHANES website, aligning with the laboratory technologists and anthropometry procedures of our previous studies [22, 23].

**Fig. 1:**
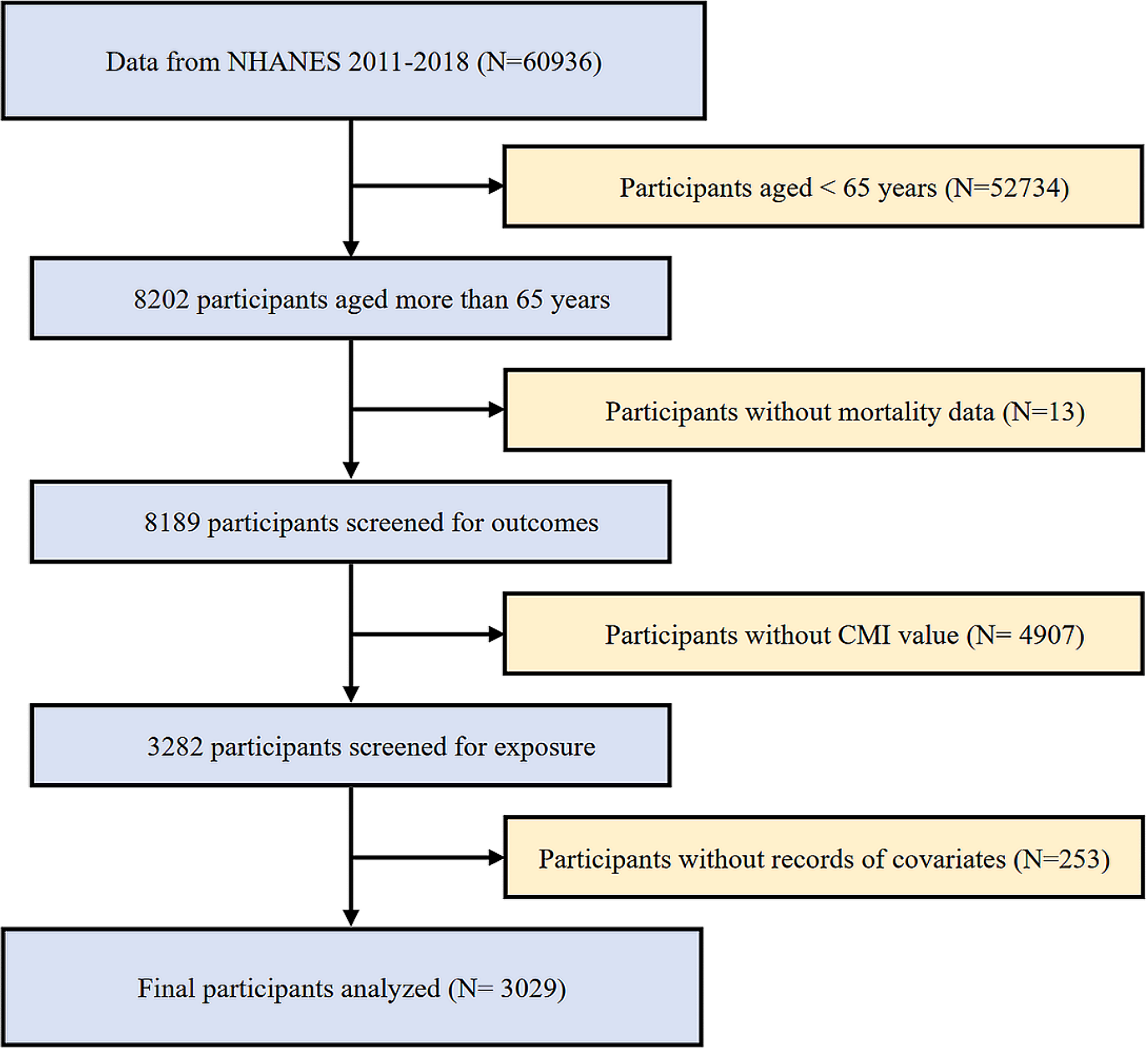
Flowchart of study participants

For the present prospective cohort study, we screened and analyzed data spanning 6 two-year cycles from 2005 to 2016. To uphold the integrity and reliability of results, the specific exclusion criteria were applied including (1) individuals<65 years of age (N=52734); (2) individuals without complete mortality data (N=13); (3) individuals without CMI value (N=4907); (4) individuals without records of necessary covariates including weight (N=6), leukocyte (N=15), drinking status (N=157), smoking status (N=4), eGFR (N=14), the history of hypertension (N=5), DM (N=8), coronary heart disease (CHD) (N=27), angina (N=9), heart attack (N=6) and stroke (N=2). A total of 3029 participants were enrolled from the years 2005 to 2016 in our study (Fig. 1).

### Assessment of CMI

The CMI was calculated using the formula:

CMI = triglyceride (TG, mmol/L) / high-density lipoprotein cholesterol (HDL-C, mmol/L) waist circumference (WC, cm)/ height (cm) [15].

CMI was treated as a continuous exposure variable in our study and all enrolled participants were stratified into tertiles based on CMI values for subsequent analyses.

### Assessment of all-cause and cardiovascular mortality

In the present study, the primary outcomes included all-cause and cardiovascular mortality. To determine the mortality status, the NHANES public-use linked mortality file as of December 31, 2019, was utilized in conjunction with the National Death Index (NDI) by the National Center for Health Statistics (NCHS) through the implementation of a probability matching algorithm. Additionally, the International Statistical Classification of Diseases, 10th Revision (ICD-10) was utilized to underly the cause of death. Cardiovascular mortality was described as death as a consequence of diseases of the heart (I00-I09, I11, I13, I20-I51) and cerebrovascular diseases (I60-I69) [24].

### Covariates

In the present study, following covariates were collected including gender, age, race, education level, family poverty-to-income ratio (PIR), body mass index (BMI), WC, waist-to-height ratio (WHtR), smoking, drinking, leukocyte, neutrophil, lymphocyte, systemic immune-inflammation index (SII), neutrophil to lymphocyte ratio (NLR), hemoglobin, platelet, albumin, total cholesterol (TC), TG, low-density lipoprotein cholesterol (LDL-C), HDL-C, creatinine, blood urea nitrogen (BUN), estimated glomerular filtration rate (eGFR), urinary albumin-creatinine ratio (UACR), hemoglobin A1c (HBA1c), hypertension, DM, cardiac disease history and stroke history.

BMI was the ratio of weight (kg) to height (m) squared. Smoking status was categorized into never, former and now according to the questionnaire "Smoked at least 100 cigarettes in life?" (SMQ020) and "Do you now smoke cigarettes" (SMQ040). Based on the questionnaire "Had at least 12 alcohol drinks/1yr" (ALQ101), drinking status was categorized into two groups based on whether participants had at least 12 drinks per year, and 1 unit of drink is equivalent to 12 ounces of beer, 5 ounces of wine, or 1.5 ounces of liquor. The chronic kidney disease epidemiology collaboration (CKD-EPI) formula was utilized to calculate the eGFR [25]. The self-reported questionnaires were used for the diagnoses of hypertension (BPD035), DM (DIQ010), heart failure (MCQ160b), CHD(MCQ160c), angina (MCQ160d), heart attack (MCQ160e) and stroke (MCQ160f).

The formulas for calculating the relevant indexes are as follows:

WHtR = WC (cm)/ height (cm),

NLR = neutrophil (/L)/lymphocyte (/L),

SII = platelet (/L) *neutrophil (/L)/lymphocyte (/L).

### Statistical analysis

All statistical analyses were performed with R software (version 4.2.2), EmpowerStats (version 2.0) along with the use of rms package and MSTATA. Categorical variables are expressed as frequencies and percentages, while continuous variables are expressed as medians and interquartile ranges. The Chi-squared test or Kruskal-Wallis H test was used to analyze various CMI tertile categories. A statistically significant result was determined as a two-sided p-value<0.05.

Multivariate Cox proportional hazard models were estimated for the associations of CMI with all-cause and cardiovascular mortality. The findings were displayed in the form of hazard ratios (HRs) and 95% confidence intervals (CI). Model 1 was unadjusted. Model 2 was modified to account for gender, age, and race. Based on Model 2, Model 3 additionally adjusted for smoking, drinking, leukocyte, hemoglobin, platelet, weight, TC, eGFR, the history of hypertension, DM, CHD, angina, heart attack and stroke. Kaplan-Meier curves were conducted to estimate survival over time progression, with the log-rank test used to assess the disparity among the various survival curves. Additionally, the multivariate logistics and Cox regression were performed in the associations between inflammatory indicators with CMI, all-cause mortality and cardiovascular mortality respectively. The inflammatory-related indicators included leukocyte, neutrophil, lymphocyte, NLR and SII. The identical statistical techniques mentioned above were also utilized in the subgroup analyses to investigate potential differences among specific populations including gender, race, education level, family PIR, hypertension, DM, smoking and drinking subgroups.

"mediation" package in R 4.2.2. was utilized to perform Mediation analysis assessing the mediating effects of inflammatory indicators (leukocyte, neutrophil, lymphocyte, NLR, and SII) on the associations of CMI with mortality, adjusted by gender, age, race, smoking, drinking, hemoglobin, platelet, weight, TC, eGFR, the history of hypertension, DM, CHD, angina, heart attack and stroke. The presence of a mediating effect was defined as satisfying all of the following conditions having a significant indirect effect, a significant total effect, and a positive proportion of the mediator effect.

## Results

### Participants characteristics

Table 1 presents the baseline characteristics of enrolled participants in the present study with different CMI tertiles. Within all enrolled participants, the CMI values were 0.74±0.66 and ranging from 0.27±0.08 in T1, 0.56±0.10 in T2, and 1.37±0.80 in T3. In comparison with those in the decreased CMI group, participants with elevated levels of CMI had an increasing proportion of males, smoking and lower education level and family PIR. In addition, participants in higher tertiles showed significantly higher levels of leukocyte, neutrophil, lymphocyte, SII and had a higher prevalence of hypertension, DM, heart failure, stroke, CHD, angina, heart attack history.

**Table 1:** Characteristics of the study population with various CMI tertiles

Characteristics	Overall (N=3029)		Tertiles of CMI			*P *value
			T1 (N=1010)	T2 (N=1009)	T3 (N=1010)	
Gender						0.001
Male	1507 (49.75%)		462 (45.74%)	501 (49.65%)	544 (53.86%)	
Female	1522 (50.25%)		548 (54.26%)	508 (50.35%)	466 (46.14%)	
Age, years	73.28±5.50		73.55±5.56	73.53±5.53	72.77±5.38	0.001
Race, n (%)						<0.001
Mexican American	321 (10.60%)		62 (6.14%)	124 (12.29%)	135 (13.37%)	
Other Hispanic	271 (8.95%)		71 (7.03%)	92 (9.12%)	108 (10.69%)	
Non-Hispanic White	1792 (59.16%)		582 (57.62%)	578 (57.28%)	632 (62.57%)	
Non-Hispanic Black	469 (15.48%)		230 (22.77%)	155 (15.36%)	84 (8.32%)	
Other Race	176 (5.81%)		65 (6.44%)	60 (5.95%)	51 (5.05%)	
Education level, n (%)						<0.001
Below high school	984 (32.49%)		278 (27.52%)	322 (31.91%)	384 (38.02%)	
High school	740 (24.43%)		212 (20.99%)	265 (26.26%)	263 (26.04%)	
Above high school	1305 (43.08%)		520 (51.49%)	422 (41.82%)	363 (35.94%)	
Family PIR						<0.001
<1.29	782 (25.82%)		215 (21.29%)	248 (24.58%)	319 (31.58%)	
1.30-3.49	1247 (41.17%)		403 (39.90%)	429 (42.52%)	415 (41.09%)	
>3.50	1000 (33.01%)		392 (38.81%)	332 (32.90%)	276 (27.33%)	
Smoking, n (%)						0.047
Never	1468 (48.46%)		515 (50.99%)	502 (49.75%)	451 (44.65%)	
Former	1265 (41.76%)		396 (39.21%)	413 (40.93%)	456 (45.15%)	
Now	296 (9.77%)		99 (9.80%)	94 (9.32%)	103 (10.20%)	
Drinking, n (%)						0.564
<12 drinks/year	1095 (36.15%)		353 (34.95%)	366 (36.27%)	376 (37.23%)	
≥12 drinks/year	1934 (63.85%)		657 (65.05%)	643 (63.73%)	634 (62.77%)	
BMI, kg/m^2^	28.63±5.78		25.83±4.88	28.61±4.99	31.44±5.99	<0.001
WC, cm	101.91±14.29		94.05±12.69	102.12±12.21	109.57±13.51	<0.001
WHtR	0.62±0.08		0.57±0.08	0.62±0.07	0.66±0.08	<0.001
Leukocyte, 10^9^/L	6.80±3.15		6.34±3.96	6.75±2.44	7.31±2.76	<0.001
Neutrophil, 10^9^/L	4.02±1.55		3.71±1.55	4.04±1.51	4.32±1.54	<0.001
Lymphocyte, 10^9^/L	1.95±2.54		1.85±3.51	1.90±1.74	2.10±1.99	<0.001
NLR	2.47±1.52		2.46±1.67	2.50±1.51	2.46±1.37	0.159
SII	567.16±395.40		557.64±429.08	570.73±389.09	573.14±365.58	0.037
Hemoglobin, g/dL	13.93±1.51		13.71±1.47	13.94±1.51	14.15±1.53	<0.001
Platelets, 10^9^/L	229.11±64.86		225.18±62.82	229.86±66.56	232.28±65.03	0.030
Albumin, g/L	41.75±2.96		41.76±2.97	41.74±2.92	41.75±3.00	0.998
TC, mmol/L	4.93±1.11		4.95±1.06	4.89±1.13	4.95±1.14	0.421
TG, mmol/L	1.44±0.81		0.82±0.24	1.28±0.32	2.22±0.90	<0.001
LDL-C, mmol/L	2.82±0.95		2.75±0.89	2.90±0.96	2.81±0.99	0.002
HDL-C, mmol/L	1.45±0.44		1.82±0.43	1.41±0.29	1.13±0.23	<0.001
Creatinine, umol/L	90.10±41.25		86.39±41.31	90.76±40.16	93.14±42.03	<0.001
BUN, mg/dl	17.00±7.35		16.50±6.28	16.88±7.54	17.63±8.06	0.077
eGFR, ml/min/1.73m^2^	70.41±18.60		72.94±17.66	69.86±18.78	68.42±19.07	<0.001
UACR, mg/g	73.42±348.93		51.72±246.30	76.41±405.72	92.12±373.52	<0.001
FBG, mmol/L	6.48±1.97		6.01±1.49	6.42±1.93	7.01±2.28	<0.001
OGTT, mmol/L	8.20±3.11		7.39±2.57	8.27±3.14	9.16±3.42	<0.001
HBA1c, %	6.05±1.04		5.82±0.77	6.06±1.12	6.27±1.14	<0.001
Fasting insulin, uU/mL	13.46±16.52		9.20±13.92	12.63±14.53	18.56±19.23	<0.001
Hypertension, n (%)	1910 (63.06%)		577 (57.13%)	628 (62.24%)	705 (69.80%)	<0.001
DM, n (%)	694 (22.91%)		148 (14.65%)	236 (23.39%)	310 (30.69%)	<0.001
Heart failure, n (%)	238 (7.88%)		61 (6.04%)	66 (6.55%)	111 (11.08%)	<0.001
CHD, n (%)	348 (11.49%)		99 (9.80%)	112 (11.10%)	137 (13.56%)	0.027
Angina, n (%)	191 (6.31%)		48 (4.75%)	66 (6.54%)	77 (7.62%)	0.025
Heart attack, n (%)	320 (10.56%)		94 (9.31%)	95 (9.42%)	131 (12.97%)	0.010
Stroke, n (%)	266 (8.78%)		79 (7.82%)	93 (9.22%)	94 (9.31%)	0.417
All-cause mortality						0.148
Yes	1015 (33.51%)		315 (31.19%)	354 (35.08%)	346 (34.26%)	
No	2014 (66.49%)		695 (68.81%)	655 (64.92%)	664 (65.74%)	
Cardiovascular mortailty						0.166
Yes	348 (11.49%)		103 (10.20%)	130 (12.88%)	115 (11.39%)	
No	2681 (88.51%)		907 (89.80%)	879 (87.12%)	895 (88.61%)	
Follow-up time (months)	89.20±42.67		85.33±41.32	90.16±43.00	92.12±43.41	0.001
CMI	0.74±0.66		0.27±0.08	0.56±0.10	1.37±0.80	<0.001

*CMI* cardiometabolic index, *PIR* poverty-to-income ratio, *BMI* body mass index, *WC* waist circumference, *WHtR* Waist-to-height ratio, *NLR* neutrophil to lymphocyte ratio, *SII* systemic immune-inflammation index, *TC* total cholesterol, *TG* triglyceride, *LDL-C* low-density lipoprotein cholesterol, *HDL-C* high-density lipoprotein cholesterol, *BUN* blood urea nitrogen, *eGFR* estimated glomerular filtration rate, *UACR* urinary albumin/creatinine ratio, *FBG* fasting blood glucose, *OGTT* oral glucose tolerance test, *HBA1c* hemoglobin A1c, *DM* diabetes mellites, *CHD* coronary heart disease

### Associations of CMI with all-cause and cardiovascular mortality

**Fig. 2:**
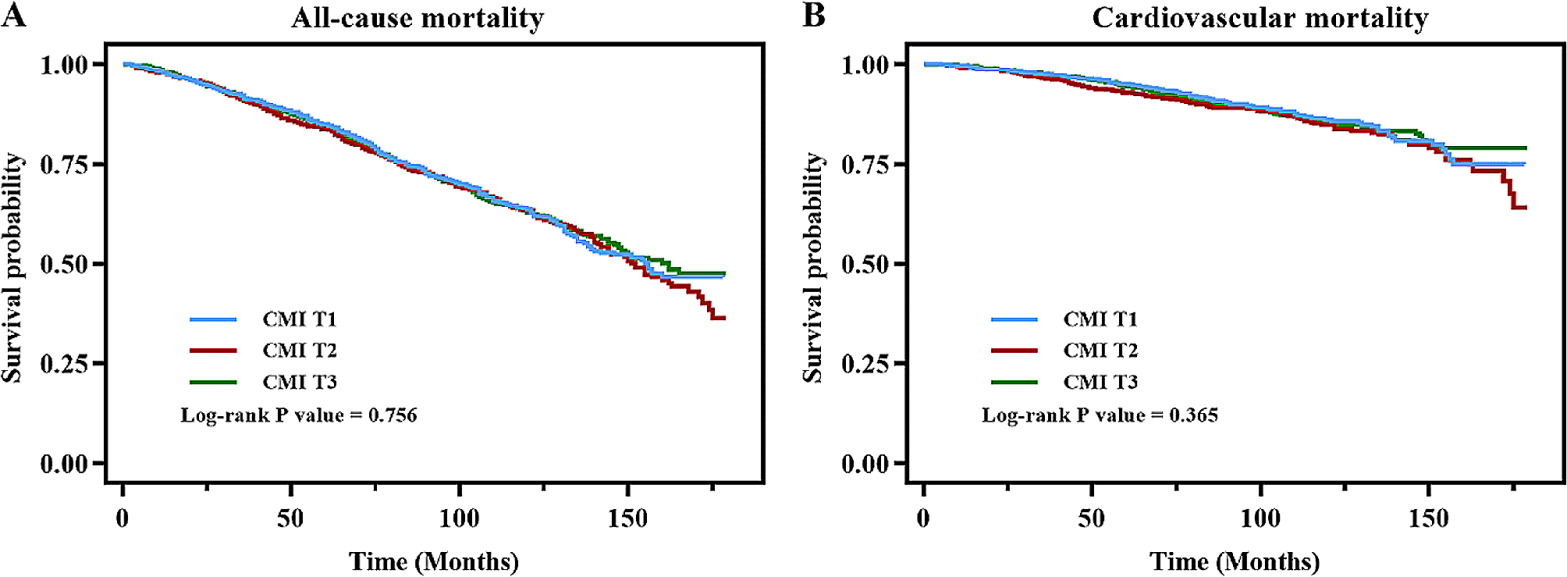
Kaplan–Meier curves of the survival rate of participants with CMI tertiles (**A** All-cause mortality, **B** Cardiovascular mortality)

During an average of 89.20 months follow-up, 1015 deaths and 348 cardiovascular-related deaths occurred in total. Table 2 displays the associations of CMI with all-cause mortality and cardiovascular mortality. In model 1, CMI had no associations with all-cause mortality and cardiovascular mortality. In model 2, CMI was positively associated with all-cause mortality (HR=1.14, 95% CI=1.05-1.24) and cardiovascular mortality (HR=1.17, 95% CI=1.01-1.35). After adjusting all interfering factors in model 3, significant associations sustained positive and significant in all-cause mortality group (HR=1.11, 95% CI=1.01-1.21). However, in fully adjusted model 3, the HRs with corresponding CIs did not show positively significant correlations among T1, T2 and T3. Besides, the Kaplan-Meier survival plots are shown in Fig. 2 and indicated that participants with CMI tertiles did not show differences in all-cause mortality (*P*=0.756) and cardiovascular mortality (*P*=0.365).

**Table 2:** The associations of CMI with all-cause mortality and cardiovascular mortality in the older adults

	HR (95% CI)		
	Model 1	Model 2	Model 3
All-cause mortality			
CMI	1.07 (0.98, 1.16)	1.14 (1.05, 1.24)	1.11 (1.01, 1.21)
Tertile 1	1	1	1
Tertile 2	1.03 (0.89, 1.19)	1.05 (0.90, 1.22)	1.02 (0.87, 1.20)
Tertile 3	0.97 (0.84, 1.13)	1.09 (0.94, 1.27)	1.07 (0.90, 1.27)
P for trend	0.596	0.270	0.451
Cardiovascular mortality			
CMI	1.05 (0.91, 1.22)	1.17 (1.01, 1.35)	1.12 (0.96, 1.30)
Tertile 1	1	1	1
Tertile 2	1.19 (0.92, 1.53)	1.24 (0.96, 1.60)	1.16 (0.88, 1.52)
Tertile 3	0.97 (0.75, 1.27)	1.17 (0.90, 1.54)	1.15 (0.86, 1.55)
P for trend	0.563	0.376	0.464

Model 1 adjust for: none

Model 2 adjust for: gender, age, race

Model 3 adjust for: gender, age, race, smoking, drinking, weight, leukocyte, hemoglobin, platelet, TC, eGFR, hypertension, DM, CHD, angina, heart attack and stroke

*CMI* cardiometabolic index, *HR* hazard ratio, *CI* confidence interval, *PIR* poverty-to-income ratio, *TC* total cholesterol, *eGFR* estimated glomerular filtration rate,* DM* diabetes mellites,* CHD* coronary heart disease

### Subgroup Analysis

**Fig. 3:**
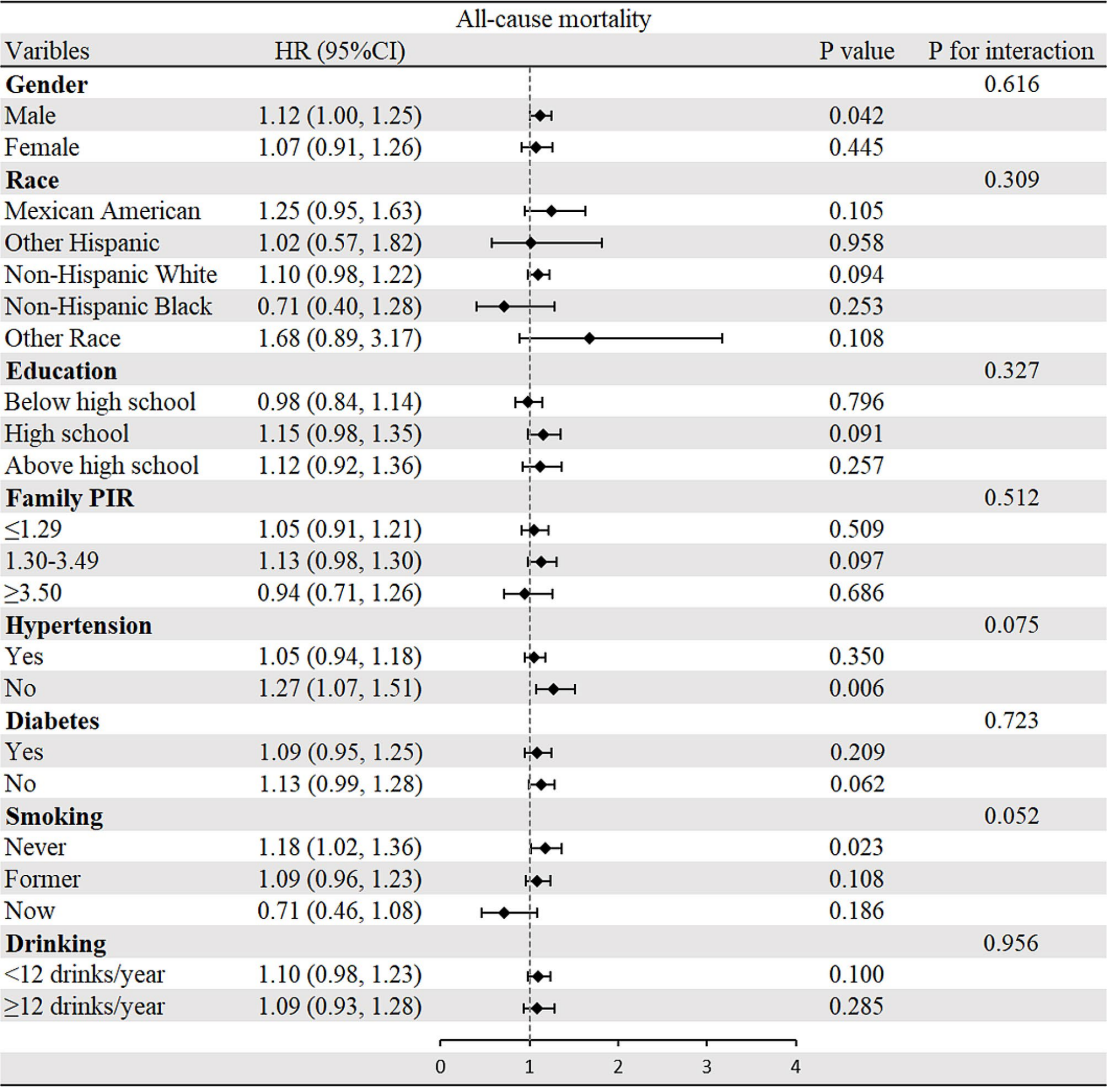
Subgroup analysis of associations between CMI and all-cause mortality

**Fig. 4:**
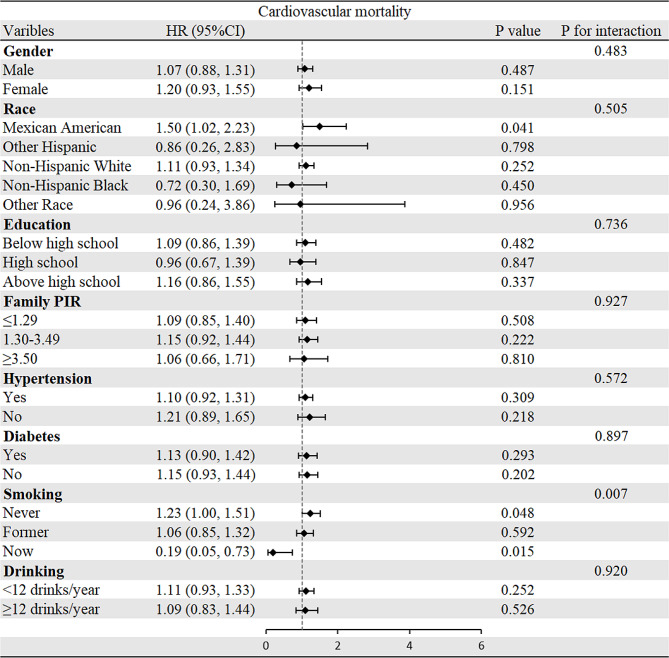
Subgroup analysis of associations between CMI and cardiovascular mortality

To evaluate the relationships of CMI with all-cause and cardiovascular mortality, subgroup analyses were performed (Figs. 3 and 4). Interestingly, most analyses did not show differences within groups except that the positive correlation between CMI and cardiovascular mortality was stronger in the participants who were never or formerly smoke. Regarding the all-cause mortality group, there was no significant difference observed in all subgroup analyses.

### Associations of inflammation with CMI and mortality

Table 3 displays the associations of CMI and inflammation-related indicators after multivariate logistic regression. After adjusting all interfering factors, CMI was positively associated with leukocyte (β=0.36, 95% CI=0.18-0.54, *P*<0.001), neutrophil (β=0.11, 95% CI=0.03-0.19, *P*=0.010), lymphocyte (β=0.20, 95% CI=0.05-0.35, *P*=0.010) and negatively associated with NLR (β=-0.14, 95% CI=-0.23--0.06, *P*=0.001) and SII (β=-29.62, 95% CI =-49.63--9.62, *P*=0.004). Cox regression models of inflammationrelated indicators with all-cause mortality and cardiovascular mortality are shown in Table 4. Most indicators were positively related to the mortality except lymphocytes with all-cause mortality and leukocytes with cardiovascular mortality.

**Table 3:** The associations between CMI and inflammation-related indicators

	β value	95% CI	*P* value
*Leukocyte*			
Model 1	0.55	0.39, 0.72	<0.001
Model 2	0.53	0.36, 0.70	<0.001
Model 3	0.36	0.18, 0.54	<0.001
*Neutrophil*			
Model 1	0.29	0.21, 0.37	<0.001
Model 2	0.25	0.17, 0.34	<0.001
Model 3	0.11	0.03, 0.19	0.010
*Lymphocyte*			
Model 1	0.19	0.06, 0.33	0.006
Model 2	0.21	0.07, 0.35	0.003
Model 3	0.20	0.05, 0.35	0.010
*NLR*			
Model 1	-0.04	-0.13, 0.04	0.282
Model 2	-0.08	-0.16, 0.0	0.053
Model 3	-0.14	-0.23, -0.06	0.001
*SII*			
Model 1	-3.35	-25.68, 16.98	0.689
Model 2	-7.28	-28.71, 14.14	0.505
Model 3	-29.62	-49.63, -9.62	0.004

Model 1 adjust for: none

Model 2 adjust for: gender, age, race

Model 3 adjust for: gender, age, race, smoking, drinking, weight, hemoglobin, platelet, TC, eGFR, hypertension, DM, CHD, angina, heart attack and stroke

*CMI* cardiometabolic index, *HR* hazard ratio, *CI* confidence interval, *PIR* poverty-to-income ratio, *TC* total cholesterol, *eGFR* estimated glomerular filtration rate, *DM* diabetes mellites, *CHD* coronary heart disease

**Table 4:** The associations of inflammation-related indicators with all-cause mortality and cardiovascular mortality

	HR (95% CI)		
	Model 1	Model 2	Model 3
*All-cause mortality*			
Leukocyte	1.03 (1.03, 1.04)	1.02 (1.02, 1.03)	1.02 (1.01, 1.03)
Neutrophil	1.20 (1.16, 1.24)	1.16 (1.11, 1.20)	1.18 (1.11, 1.20)
Lymphocyte	1.02 (0.99, 1.04)	1.01 (1.00, 1.03)	1.01 (0.99, 1.03)
NLR	1.19 (1.16, 1.21)	1.13 (1.10, 1.16)	1.11 (1.09, 1.14)
SII	1.00 (1.00, 1.00)	1.00 (1.00, 1.00)	1.00 (1.00, 1.00)
*Cardiovascular mortality*			
Leukocyte	1.02 (1.00, 1.05)	1.01 (0.99, 1.04)	1.01 (0.98, 1.03)
Neutrophil	1.17 (1.10, 1.25)	1.13 (1.06, 1.21)	1.17 (1.09, 1.25)
Lymphocyte	0.65 (0.54, 0.78)	0.79 (0.66, 0.95)	0.83 (0.69, 0.99)
NLR	1.19 (1.15, 1.23)	1.13 (1.08, 1.18)	1.11 (1.07, 1.16)
SII	1.00 (1.00, 1.00)	1.00 (1.00, 1.00)	1.00 (1.00, 1.00)

Model 1 adjust for: none

Model 2 adjust for: gender, age, race

Model 3 adjust for: gender, age, race, smoking, drinking, weight, hemoglobin, platelet, TC, eGFR, hypertension, DM, CHD, angina, heart attack and stroke

*CMI* cardiometabolic index, *HR* hazard ratio, *CI* confidence interval, *PIR* poverty-to-income ratio, *TC* total cholesterol, *eGFR* estimated glomerular filtration rate, *DM* diabetes mellites, *CHD* coronary heart disease

### Mediating role of inflammationrelated indicators

**Fig. 5:**
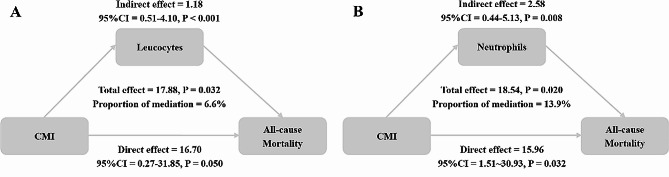
Analysis of the mediation by leukocytes (**A**) and neutrophils (**B**) of the associations of CMI with all-cause mortality

Fig. 5 shows that leukocyte mediated 6.6% of the association between CMI and all-cause mortality. Regarding the analysis of neutrophils, the proportion of mediation was 13.9%. Additionally, we also assessed the mediating roles of other inflammatory indicators including lymphocytes, NLR, and SII (Appendix Table S1).

## Discussion

In our study, we unveiled a positive association of CMI with mortality among the older adults and the findings persisted significantly in the comprehensively adjusted model. After the positive correlations of inflammation-related indicators with mortality and CMI were demonstrated, mediation analysis was performed and highlighted the significant roles of leukocytes and neutrophils in linking CMI with all-cause mortality, proposing inflammation as a potential underlying mechanism in these associations. Therefore, monitoring CMI among the older population offers a simple yet potent approach for epidemiological studies on worse prognosis.

CMI, as an innovative anthropometric index, was initially introduced by Wakabayashi et al. in 2015 to identify DM, demonstrating its significant correlation with hyperglycemia [15]. Subsequent research further explored the association between CMI and DM among Japanese adults (HR=1.65) and identified a non-linear relationship with an inflection point at a CMI of 1.01 [26]. Similarly, Qiu et al. reported that individuals with elevated CMI had a considerably increased risk of new-onset DM (HR=1.78), and those with initially low CMI who shifted to high CMI during follow-up saw their risk of developing DM elevated by 75% [27]. Moreover, researches have not only confirmed CMI’s linkage to increased risks of new-onset DM but also expanded its relevance to CVD and other metabolic disorders. Cai et al. found a positive association between CMI and the onset risk of CVD in hypertension and obstructive sleep apnea patients (HR=1.31) [17]. Higashiyama et al. discovered a higher risk for ischemic CVD in participants without MetS who had higher CMI [28]. Lazzer et al. indicated that CMI had higher sensitivity and specificity in detecting MetS compared with other anthropometric indexes [18]. Radetti et al. revealed the validity of CMI in identifying MetS in severely obese children and adolescents (AUC=0.8476) [29]. Zou et al. identified a positive association between CMI and elevated risk of NAFLD in the general population (AUC=0.8359) [16]. Liu et al. observed that CMI was a potent predictor of NAFLD in Chinese women (AUC=0.833) [30]. Miao et al. suggested CMI as an independent risk factor for albuminuria (Odds Ratio=1.160), highlighting its potential in predicting renal dysfunction [31]. Numerous studies showed that CMI was related to various systematic diseases, underlining its association with worse prognosis. However, there is no prior study to evaluate the associations CMI with long-term mortality, especially in the older adults with higher risk of comorbidities.

Other anthropometric and metabolic indices such as triglyceride glucose (TyG) index, WHtR as well as atherogenic index of plasma (AIP)—each validated as being associated with higher long-term mortality. Chen et al. elucidated a positive association of the TyG index with all-cause (HR=1.160) and cardiovascular mortality (HR=1.213) in the overall population [32]. Wang et al. suggested that the TyG index was a promising indicator for predicting all-cause mortality (HR=3.64) in patients younger than 65 years old with CVD [33]. Zhang et al. observed U-shaped associations of the TyG index with all-cause and cardiovascular mortality in CVD patients with DM or pre-DM and the threshold TyG index values were 9.05 and 8.84 in all-cause mortality and cardiovascular mortality [34]. Shen et al. demonstrated that the TyG index was related to mortality (HR=1.44) in ACS patients with DM aged over 80 years old [35]. Similarly, Tamosiunas et al. suggested AIP was positively associated with all-cause mortality among women (HR=1.36) and cardiovascular mortality among men (HR=1.40) [36]. Duiyimuhan et al. demonstrated that AIP was associated with both all-cause and cardiovascular mortality in patients with hypertension and the U-shape associations were observed with RCS curves [37]. Considering of WHtR, Chen et al. suggested that WHtR was a predictive factor of all-cause death (HR=1.96) in suffering from heart failure with preserved ejection fraction [38]. Meta-analysis revealed that in the overall population, the HR for all-cause mortality increased by 16% and cardiovascular mortality by 19% with every unit of continuous WHtR measurements increased [39]. In the present study, we introduced CMI as a novel predictor of all-cause mortality in the older adults. To date, the current study is the first study to evaluate the prognostic value of CMI in the older population, as a metabolism-related index easy to obtain, further studies are necessary to perform for the validation of CMI in public health monitoring of other specific populations.

Although CMI is highly related to all-cause mortality as elucidated by our study, the underlying biological mechanisms driving these associations are not fully deciphered. Chronic inflammation is thought to be a significant factor in the progression of CVD and DM, which may explain the positive association between CMI and poor prognosis. Chronic low-grade inflammation is a well-documented contributor to insulin resistance and hyperglycemia, leading to the onset of DM and the following development of macrovascular and microvascular complications [9, 10]. The leading pathophysiological processes to explain insulin resistance and T2D include oxidative stress, amyloid deposition in the pancreas, endoplasmic reticulum stress, among others. Intriguingly, these cellular stressors can either trigger an inflammatory reaction or be worsened by or linked to inflammation [10, 40, 41]. Additionally, inflammation has been elucidated as a crucial contributor to atherosclerosis, resulting in the progress of CVD [11, 12]. Inflammatory cells also have important roles in the formation of plaque [12]. Thus far, every type of inflammatory cell has been discovered in atherosclerotic lesions taken from both experimental models and patients [42, 43]. In the early stage of atherosclerosis, macrophage subgroups were seen as the initial cells to infiltrate arterial lesions and transform into culprit foam cells [44]. Neutrophils and lymphocytes (including T and B cells) were subsequently identified and associated with the vulnerability and rupture risk of plaque [45, 46]. Additionally, recent clinical trials highlighted the efficacy of anti-inflammatory therapies including colchicine and canakinumab in secondary prevention of cardiovascular afflictions [12, 47]. Moreover, as individuals age, their immune system undergoes alterations that eventually lead to a noticeable and severe decline, resulting in increased rates of mortality from infectious and long-term comorbidity [48]. Inflammation can predict all-cause mortality beyond established risk factors in the older adults [13, 14]. Varadhan et al. suggested that inflammatory indicators including C-reactive protein, and interleukin-6, among others were independent predictors of 5-year mortality in the older population [13]. Moreover, Different inflammation indicators may correspond to various pathways. The signaling pathways involved in the regulation of various leukocyte functions includes NADPH Oxidase Pathway, MAPK Signaling Pathways, PI3K/Akt Pathway, TCR Pathway, BCR Pathway and NF-κB Signaling Pathway. Neutrophils, a pivotal class of leukocytes, play a fundamental role in the innate immune response. These cells are equipped with a variety of signaling pathways that regulate their activation, chemotaxis, phagocytosis, and the release of antimicrobial factors. Upon stimulation, neutrophils activate the NADPH oxidase Pathway, which is critical for the respiratory burst that produces reactive oxygen species (ROS) [49]. This process is essential for the microbial killing capabilities of neutrophils. The MAPK pathways, including p38, ERK, and JNK, are activated in neutrophils by various stimulation such as cytokines and microbial products. These pathways regulate a range of functions including gene expression, apoptosis, and the production of inflammatory cytokines [50]. PI3K/Akt Pathway promotes cell survival by inhibiting apoptotic processes and is also involved in mediating responses to chemokines and other inflammatory stimulation [51]. Lymphocytes, which include T cells, B cells, and natural killer cells, are central to the adaptive immune response and also play roles in innate immunity. They are regulated by complex signaling pathways that govern their development, activation, differentiation, and effector functions. The main signaling pathway involved in lymphocyte function is TCR pathway. Upon antigen recognition, the TCR engages with a peptide presented by the major histocompatibility complex (MHC) on antigen-presenting cells. This interaction initiates a cascade involving the phosphorylation of the immunoreceptor tyrosine-based activation motifs (ITAMs) by Lck and Fyn, which are Src family tyrosine kinases. This leads to the recruitment and activation of ZAP-70, further propagating the signal through downstream effectors such as Linker for Activation of T cells (LAT) and SLP-76, culminating in the activation of transcription factors NF-AT, NF-κB, and AP-1 [52, 53]. These factors drive gene expression crucial for T cell activation, proliferation, and cytokine production. In addition, similar to TCR, the BCR pathway is activated when the receptor binds its specific antigen. This results in the activation of Src family kinases like Lyn, which phosphorylate ITAMs on the Igα and Igβ chains of the BCR. This triggers a series of phosphorylation events involving Syk kinase and adaptor proteins such as BLNK, leading to the activation of several downstream pathways including PI3K/Akt, Bruton's tyrosine kinase (Btk), and PLCγ2. These pathways are essential for B cell survival, proliferation, differentiation into plasma cells, and antibody production [54]. In both T cells and B cells, the NF-κB pathway plays a crucial role in regulating immune responses by controlling the transcription of genes involved in cell survival, proliferation, and inflammatory responses. Activation of NF-κB can be triggered by TCR/BCR signaling as well as by other receptors such as Toll-like receptors and TNF receptors [53, 55]. In the present study, we hypothesized that inflammation would have significant mediating effects in the association of CMI with mortality in the older adults. In the present study, leukocytes and neutrophils mediated 6.6% and 13.9% of the association between CMI and all-cause mortality. Our findings confirm the significant mediating role of inflammation, providing validate evidence for its involvement in this association.

A major strength of our study is that the present study is a prospective cohort study, utilizing the large and representative NHANES database, which strengthen the generalizability of our findings. We provide additional evidence supporting the positive associations of CMI with all-cause mortality in the older adults. Our study also highlights the potential of CMI as an easily obtainable anthropometric index for identifying individuals with worse prognoses and underscores the mediating role of inflammation in these associations.

### Study limitations

Despite adjusting for several confounders, unmeasured or residual confounding cannot be fully excluded. Treatment variables that might influence CMI, such as fibrates, statins, and specific oral antidiabetic medications, were not considered. CMI, as a novel anthropometric index, there is few studies to evaluate different comorbidities in older adults in predicting mortality. Thus, it is unknown that whether CMI contributes to the increased mortality through various comorbidities. Besides, it is noted that the diagnosis of hypertension, DM, heart failure, coronary heart disease, angina, heart attack and stroke in this study were obtained through participant questionnaires, which may introduce recall bias, potentially affecting the accuracy of the diagnoses.

## Conclusion

The present study provided evidence for the positive associations of CMI with all-cause mortality in the older adults, while also highlighting the significant mediating role of inflammation in this relationship. These insights added to the growing evidence supporting the clinical utility of CMI in predicting worse prognosis, contributing valuable perspectives for early risk stratification and the development of intervention strategies in the older populations.

### Electronic supplementary material

Below is the link to the electronic supplementary material.

**Additional file 1: Table S1**. Analysis of the mediation by inflammation-related indicators of the associations of CMI with all-cause mortality and cardiovascular mortality.

## Data Availability

No datasets were generated or analysed during the current study.

## Abbreviations

AIP: Atherogenic Index of Plasma
BMI: Body mass index
BUN: Blood urea nitrogen
CI: Confidence interval
CMI: Cardiometabolic index
CVD: Cardiovascular disease
DM: Diabetes mellitus
eGFR: Estimated glomerular filtration rate
FBG: Fasting blood glucose
HbA1c: Hemoglobin A1c
HDL-C: High-density lipoprotein cholesterol
HR: Hazard ratio
LDL-C: Low-density lipoprotein cholesterol
METS: Metabolic syndrome
NAFLD: Nonalcoholic fatty liver disease
NHANES: National Health and Nutrition Examination Survey
NLR: Neutrophil to lymphocyte ratio
OGTT: Oral glucose tolerance test
family PIR: family poverty-to-income ratio
SII: Systemic immune-inflammation index
TC: Total cholesterol
TG: Triglyceride
TyG index: Triglyceride glucose index
UACR: Urinary albumin/creatinine ratio
WC: Waist circumference
WHtR: Waist-to-height ratio
ROS: Reactive oxygen species
MHC: Major histocompatibility complex
