# Supplementary material for: Is systemic inflammation a missing link between cardiometabolic index with mortality? Evidence from a large population-based study
Source: Cardiovasc Diabetol. 2024 Jun 20;23:212. doi: 10.1186/s12933-024-02251-w (PMC11191290; doi:10.1186/s12933-024-02251-w)
Supplement: Supplementary file 1 — Additional file 1: Table S1. Analysis of the mediation by inflammation-related indicators of the associations of CMI with all-cause mortality and cardiovascular mortality. [file 12933_2024_2251_MOESM1_ESM.docx]

**Table S1** Analysis of the mediation by inflammation-related indicators of the associations of CMI with all-cause mortality and cardiovascular mortality

|  | **Mediation effect (95% CI), P value** | | |  |
| --- | --- | --- | --- | --- |
|  | Total effect | Indirect effect | Direct effect | Mediation |
| **All-cause mortality** |  |  |  |  |
| Leukocytes | 17.88 (2.12, 33.81) 0.032 | 1.18 (0.51, 4.10) <0.001 | 16.70 (0.27, 31.85) 0.050 | 6.6% |
| Neutrophils | 18.54 (4.35, 34.31) 0.020 | 2.58 (0.44, 5.13) 0.008 | 15.96 (1.51, 30.93) 0.032 | 13.9% |
| Lymphocytes | 18.17 (4.12, 33.17) 0.020 | 0.28 (-1.96, 1.04) 0.420 | 17.90 (3.71, 33.53) 0.020 | 1.5% |
| NLR | 18.65 (5.76, 34.01) 0.016 | -2.63 (-4.76, -1.31) <0.001 | 21.27 (8.20, 36.40) 0.008 | -14.1% |
| SII | 18.40 (5.35, 33.64) 0.016 | -2.28 (-4.21, -1.00) <0.001 | 20.69 (7.93, 36.12) 0.012 | -12.4% |
| **Cardiovascular mortality** |  |  |  |  |
| Leukocytes | 34.84 (-30.82, 90.03) 0.208 | 0.51 (-2.58, 5.84) 0.572 | 34.33 (-31.89, 87.86) 0.224 | 1.5% |
| Neutrophils | 38.59 (-13.16, 90.49) 0.152 | 4.96 (0.69, 11.22) 0.008 | 33.62 (-20.02, 85.69) 0.220 | 12.9% |
| Lymphocytes | 33.01 (-23.67, 94.46) 0.240 | -14.13 (-50.47, -2.05) 0.004 | 47.15 (-6.13, 118.85) 0.092 | -42.8% |
| NLR | 37.93 (-11.66, 87.02) 0.128 | -4.85 (-9.68, -2.26) <0.001 | 42.78 (-5.57, 91.37) 0.096 | -12.8% |
| SII | 37.28 (-12.51, 87.21) 0.148 | -4.09 (-8.82, -1.80) <0.001 | 41.37 (-6.86, 90.34) 0.104 | -11.0% |

Adjust for: gender, age, race, smoking, drinking, weight, hemoglobin, platelet, TC, eGFR, hypertension, DM, CHD, angina, heart attack and stroke

Abbreviation: CMI cardiometabolic index, CI confidence interval, NLR neutrophil to lymphocyte ratio, SII systemic immune-inflammation index, TC total cholesterol, eGFR estimated glomerular filtration rate, DM diabetes mellites, CHD coronary heart disease
